# Disrupted circadian control promotes oncogenesis in breast cancer

**DOI:** 10.6026/973206300214945

**Published:** 2025-12-15

**Authors:** Yasmin Fatima, Mohammad Kashif, Prashant Ankur Jain

**Affiliations:** 1 Department of Computational Biology and Bioinformatics, Sam Higginbottom Institute of Agriculture, Technology and Sciences, Prayagraj, Uttar Pradesh, India; 2 Department of Life Sciences, Parul Institute of Applied Sciences, Parul University, Vadodara, Gujarat, 391760, India

**Keywords:** Circadian rhythm, breast cancer, BMAL1, CLOCK, PER3, PI3K-Akt signalling, hub genes, transcriptomics, systems biology, network analysis

## Abstract

Breast cancer progression is increasingly linked to disturbances in circadian rhythm genes, although the underlying molecular mechanisms remain poorly understood. Circadian rhythm genes help maintain normal biological processes and their disruption contributes to breast cancer development. Transcriptomic data from breast cancer (MCF-7) and normal breast (MCF-10A) cell lines from the GSE76370 dataset were analyzed using the limma R package to identify differentially expressed genes. Functional enrichment and network analyses using GO, KEGG, STRING and Cytoscape revealed 1,788 DEGs, including 1,008 upregulated genes involved in DNA replication, chromatin remodeling and PI3K-Akt signaling and 780 downregulated genes associated with cell adhesion and apoptosis. Disrupted expression of core circadian genes (BMAL1, CLOCK and PER3) and hub genes such as ACTB, GAPDH and CDK1 suggests that circadian gene dysregulation promotes breast cancer progression and represents a potential therapeutic target.

## Background:

Breast cancer stands as the second most prevalent cancer globally, posing a significant health challenge for millions of women worldwide. Modern lifestyle-induced circadian disruption, especially night-shift work and artificial light exposure, impairs neuroendocrine regulation and increases risk for endocrine-related cancers such as breast cancer [1]. While a majority of cases are estrogen receptor (ER)-positive, approximately 25-30% are ER-negative [2, 3]. Circadian disruption from light-at-night, shift work and misaligned schedules promotes tumor development and alters treatment responses, underscoring its significant impact on cancer progression and survivorship [4]. Current therapeutic strategies often hinge on biomarkers like ER, progesterone receptor (PGR) and HER2 protein expression [5]. Research has intensely focused on unraveling breast cancer's genetic roots and developing effective treatments [6, 7]. Bioinformatics, with its computational prowess, has been pivotal in deciphering breast cancer's molecular intricacies, offering fresh insights and potential treatment targets [8]. Rooted in genetic alterations, breast cancer disrupts genes crucial for cell growth, proliferation and differentiation. Deciphering these genetic causes is pivotal for understanding breast cancer's mechanisms and progression [9]. By pinpointing genes and their functions, researchers illuminate breast cancer's molecular drivers, paving the way for targeted therapies. In recent years, bioinformatics has burgeoned in breast cancer research, harnessing computational methods to dissect vast genomic and transcriptomic datasets [10]. This approach reveals genetic anomalies like somatic mutations, copy number variations and gene expression shifts that fuel breast cancer. Moreover, bioinformatics integrates diverse data, including clinical, genomic and proteomic, to construct comprehensive breast cancer profiles [11, 12]. A cornerstone of breast cancer bioinformatics lies in gene ontology and regulatory network analysis. Gene ontology categorizes genes by function, process and cellular location, unveiling their roles in breast cancer [13, 14]. Meanwhile, regulatory network analysis unveils key genes and their interactions, illuminating breast cancer's complex molecular networks [15, 16]. This research offers a thorough exploration of breast cancer, emphasizing its genetic origins and the pivotal role of bioinformatics [17, 18, 19-20]. We delve into contemporary bioinformatics approaches, such as multi-omics integration and gene ontology and regulatory network analyses [21, 22-23]. Recent advances in bioinformatics have enabled diverse applications ranging from structure-based drug discovery to immunoinformatics-driven epitope prediction, demonstrating the expanding role of computational tools in biomedical research [24, 25]. Systems biology approaches have greatly advanced our understanding of cancer complexity by integrating network theory, dynamic modeling and regulatory mechanisms. Network-based analyses, stress signal propagation models and transcriptional control studies have together revealed key insights into cancer-associated gene regulation and cellular homeostasis [26, 27-28]. This study highlights how circadian rhythm disruption contributes to breast cancer by altering key molecular pathways and hub genes. Therefore, it is of interest to investigate how disruption of circadian rhythm genes alters molecular pathways and regulatory networks involved in breast cancer progression using integrative bioinformatics approaches.

## Materials and Methods:

## Data collection:

This study involved accessing breast cancer datasets from the Gene Expression Omnibus (GEO) [29], a distinguished repository curated by the National Center for Biotechnology Information (NCBI) [30, 31]. The dataset chosen for analysis was GSE76370 [2], comprising 16 cell lines, including 8 cancerous and 8 non-cancerous variants. Selection of this dataset was driven by its significance in breast cancer investigation and its inclusive representation of malignant and non-malignant breast cell lines [7, 32]. Access to the dataset was facilitated via the GEO2R database, accessible at the URL: https://www.ncbi.nlm.nih.gov/geo/geo2r/

## Meta-analysis of the datasets:

Figure 1 (see PDF) outlines the methodology employed in this study's integrated analysis. The meta-analysis utilized the max p-value method, applying a Benjamini Hochberg false discovery rate (FDR) threshold of less than 0.001 [33]. This enabled the identification of statistically significant differentially expressed genes (DEGs) between cancerous and non-cancerous cell lines. The analysis leveraged the limma R package for its robust functionalities [34]. DEGs were identified based on criteria such as a log2 fold change (log2FC) greater than 2 and an adjusted p-value less than 0.001.

## Gene ontology analysis:

To explore the functional traits and potential pathways linked with the differentially expressed genes (DEGs), we performed gene ontology (GO) annotation and KEGG pathway enrichment analyses [35]. Information regarding GO annotation and KEGG pathway enrichment of the DEGs was sourced from the Database for Annotation, Visualization and Integrated Discovery (DAVID), accessible at https://davidbioinformatics.nih.gov/ [36]. This online platform offers extensive and current functional annotation and pathway data for the DEGs [37]. Statistical significance was determined based on a p-value threshold of less than 0.001 for the ontology terms retrieved from DAVID [38].

## Protein-protein interaction network construction:

In this study, network analysis was instrumental in revealing the regulatory roles of genes through the utilization of protein-protein interaction (PPI) data. The STRING database, accessible at https://string-db.org/ was employed to construct the PPI network of differentially expressed genes (DEGs) [39]. This widely utilized database facilitated the exploration of interactions among the DEGs, offering valuable insights into their functional associations and potential regulatory connections. The identified DEGs were inputted into the STRING database for PPI network generation, thereby creating a comprehensive network depiction. To aid in gene network visualization and subsequent analysis for identifying key regulators, the generated PPI network was further imported into the Cytoscape software [40]. Within Cytoscape, various plugins such as CytoHubba and CytoNCA were utilized, facilitating the identification of crucial regulators within the gene network.

## Network analysis:

To examine the network's topological features derived from the protein-protein interaction (PPI) data, we utilized the Cytohubba plugin network analyzer within Cytoscape 3.9.1 [40]. This robust tool enabled us to delve into the structural attributes of the network and pinpoint key nodes using various topological metrics. Additionally, to compute EigenVector values, we employed the CytoNCA plugin, offering insights into the relative significance and centrality of individual nodes within the PPI network. For identifying modules within the primary network, we utilized the "leading eigenvector method" algorithm via the MCODE plugin in Cytoscape. This algorithm facilitated the detection of densely connected regions or functional clusters within the PPI network based on default parameters.

## Key regulator gene identification:

To pinpoint key regulator genes (KR) within the breast cancer gene regulatory network, we utilized the Cytohubba plugin in Cytoscape. This robust tool enabled us to evaluate various topological aspects of the network and select the top ten genes based on degree, closeness centrality, betweenness centrality, Bottleneck, MCC (Maximal Clique Centrality) and MNC (Maximum Neighborhood Component). By integrating these diverse topological metrics, our goal was to identify key regulator genes that wielded significant importance and influence within the network. To enhance the reliability of our findings, we refined the selection process by identifying genes common across all six topological properties and get result in graphical form using SRplot online platform [41]. Specifically, we traced these common genes to the top ten hub genes, representing those with the highest degrees within the network.

## Results and Discussion:

## Differential and disrupted circadian transcriptomes distinguish cancerous MCF 7 cells from non cancerous MCF 10A cells:

The updated analysis again demonstrates substantial transcriptional differences between the cancerous and non cancerous breast cell lines. Hundreds of genes exceed the ±2 log2 fold change and adjusted p value < 0.05 thresholds, with roughly equal numbers of up and down regulated transcripts (Figure 1A - see PDF). The cancerous MCF 7 cells over express many structural and proliferation associated genes, including ribosomal proteins (RPL15, RPL30), keratins (KRT5, KRT14, KRT15) and transcripts linked to stress responses (SFN) and cell surface signalling (TACSTD2, ID3). In contrast, the non cancerous MCF 10A cells preferentially express genes involved in metabolism and differentiation, such as CXCL14, CYP3A5, SLC18A3, ALDH1A1, SPNS2 and VGF, as well as several genes linked to immune and extracellular matrix functions (IL6, SPP1, CSPG2, BEX2). These patterns highlight that tumour cells up regulated genes promoting growth and structural remodelling while repressing genes associated with differentiation, metabolism and immune signalling, underscoring the broad transcriptional reprogramming associated with malignancy.

## Circadian disruption and oncogenic transcriptional programmes distinguish MCF-7 breast cancer cells from non-cancerous MCF-10A cells:

In addition to identifying broad functional categories, a closer examination of the enrichment results in Figure 3 (see PDF) provides further insight into the biological programmes activated in the cancerous MCF 7 cells. Several biological processes exhibit exceptionally high fold enrichment, with protein localisation and megakaryocyte differentiation showing fold enrichments > 20 (Figure 2A - see PDF). These large values indicate that genes involved in intracellular trafficking and platelet like differentiation are vastly over represented among up regulated transcripts. Processes linked to DNA synthesis such as DNA replication and DNA replication chromatin organisation is enriched more than ten fold, underscoring the proliferative phenotype of MCF 7. Mitotic cell cycle and cell differentiation terms rank high in both enrichment and gene count, suggesting that the cancerous cells not only divide more rapidly but also activate developmental programmes that are dormant in non transformed epithelial cells. The presence of multicellular organism development and circulatory system development among significant terms hints at aberrant activation of embryonic pathways. Protein binding, ATP binding and identical protein binding show high gene counts and significant enrichment, consistent with increase protein synthesis and turnover (Figure 3A - see PDF). Molecular function terms such as protein binding, ATP binding and ubiquitin ligase binding suggest up regulated protein synthesis, turnover and signal transduction. Cellular component enrichment points to increased activity in compartments associated with secretion, membranes and organelles, including vesicles, endoplasmic reticulum, lysosomes, cytosol and nucleus. Enrichment of "ubiquitin protein ligase binding" points to heightened ubiquitin mediated proteolysis, while "actin binding" and "cadherin binding" suggest cytoskeletal remodelling. The presence of "DNA helicase activity", "protease binding" and "NAD binding" links up regulated genes to DNA replication, protease inhibition and redox metabolism, respectively. The cellular component analysis shows that up regulated genes localise to diverse compartments, ranging from the synaptic vesicle membrane and presynaptic membrane to the endoplasmic reticulum lumen, lysosome, spindle apparatus and focal adhesion complexes (Figure 3B - see PDF). High fold enrichment of "extracellular exosome", "extracellular region" and "extracellular matrix" indicates increased secretion and extracellular matrix interactions in MCF 7, while enrichment of nuclear and chromosomal terms reflects elevated DNA replication and transcriptional activity. KEGG pathway enrichment (Figure 3C - see PDF) highlights multiple metabolic pathways (glycolysis/gluconeogenesis, carbon metabolism, galactose metabolism) alongside cancer associated signalling networks. Elevated fold enrichment for "PI3K-Akt", "Wnt", "Hippo", "JAK-STAT" and "HIF 1" signalling reflects activation of pathways that promote growth, survival and angiogenesis in tumours. Importantly, the "p53 signalling pathway" is also enriched, indicating a transcriptional response to stress or DNA damage (Figure 3D - see PDF).

## Suppressed differentiation and adhesion genes in MCF 7 breast cancer:

Expanding further on the down regulated gene ontology and pathway analysis strengthens the narrative of how loss of specific biological programmes contributes to the malignant phenotype of MCF 7 cells. The biological process enrichment results indicate that the suppression of differentiation pathways is not confined to keratinocyte lineage commitment but extends across epidermal and epithelial development. High fold enrichment values (> 4) for "epidermis development", "skin development", "epithelial cell differentiation" and "programmed cell death" reflect a broad down regulation of genes involved in maintaining epithelial identity and executing apoptosis (Figure 4A - see PDF). The significant over representation of "cell population proliferation" and "positive regulation of apoptotic process" among down regulated genes suggests that tumour cells down regulate negative regulators of proliferation and pro apoptotic factors, thereby tipping the balance toward unchecked growth. The molecular function category underscores that MCF 7 cells down regulate multiple types of receptor and ligand binding. Enrichment of "death receptor binding" and "growth factor binding" implies reduced transcription of ligands and receptors that would normally modulate cell survival and growth, possibly conferring resistance to extrinsic apoptotic signals. The large bubble sizes for "protein binding" and "protein kinase binding" point to widespread down regulation of signalling intermediates, while "calcium ion binding" and "calcium dependent protein binding" implicate alterations in calcium mediated signaling (Figure 4B - see PDF). In the cellular component category, the pronounced enrichment of "cell-cell junction", "adherens junction", "focal adhesion" and "actin cytoskeleton" terms among down regulated genes highlights a coordinated suppression of adhesion complexes and cytoskeletal organisation. These structures are essential for maintaining tissue architecture and for restraining cell movement. Their down regulation may facilitate epithelial-mesenchymal transition and increase invasive potential. The concurrent enrichment of "extracellular exosome" and "extracellular space" suggests that secretion and extracellular matrix interactions are also altered, possibly affecting cell-+cell communication and metastatic niche preparation (Figure 4C - see PDF). The KEGG pathway analysis integrates these findings at a systems level. Down regulated genes populate pathways such as "adherens junction", "focal adhesion" and "cytotoxicity", reinforcing the disruption of cell adhesion and immune mediated cell death. Enrichment of "p53 signalling pathway" among down regulated genes may indicate inactivation of p53 mediated transcriptional responses, which ordinarily promote cell cycle arrest and apoptosis in response to DNA damage. Similarly, the presence of "NOD signalling pathway" and "Hippo signalling pathway" suggests reduced innate immune and growth control signalling. The inclusion of "lipid and atherosclerosis" and "proteoglycans in cancer" pathways among the suppressed categories hints at altered lipid metabolism and extracellular matrix composition, both of which are increasingly recognised as hallmarks of cancer progression (Figure 4D - see PDF). Taken together, these expanded results emphasize that the transcriptional programme lost in MCF 7 cells encompasses differentiation, adhesion, immune signalling and apoptosis. When juxtaposed with the up regulated pathways that promote proliferation and metabolism (Figure 4 - see PDF), it becomes clear that breast cancer cells achieve their malignant phenotype through both activation of oncogenic networks and concurrent suppression of pathways that would otherwise maintain epithelial homeostasis and restrain.

## Integrated centrality analysis of the PPI network identifies ACTB, GAPDH and CDK1 as consensus hub genes in breast cancer cells:

Comprehensive PPI network analysis revealed that the differentially expressed genes do not operate in isolation but form a densely interconnected network (Figure 5A - see PDF). To pinpoint key drivers within this network, we assessed node importance using eight complementary centrality measures. The intersection analysis (Figure 5B - see PDF) showed that three genes ACTB (β actin), GAPDH and CDK1 were consistently identified as hubs by all eight-centrality metrics, indicating that they occupy central positions in the network irrespective of the metric used. Several other genes were selected by multiple metrics, but only these three were unanimously highlighted. Ranking of the top ten hub genes across metrics (Figure 5C - see PDF) further emphasized the dominance of ACTB, GAPDH and CDK1, which held top positions for most centrality measures. The Venn diagram (Figure 5D - see PDF) focusing on eigenvector, betweenness, closeness and degree centrality confirmed an eight gene overlap; this consensus set includes ACTB, GAPDH, CDK1, FN1, CDC42, IL6, CDH1 and UBC. Visualization of these hub genes in subnetworks (Figure 5E-F - see PDF) revealed two tightly connected modules. The first module (E) comprises metabolic enzymes (ACO2, GAPDH), stress response proteins (HSP90AB1), adhesion molecules (CDH1), signalling proteins (CDC42, IL6) and metabolic co factors (GART, AKR1B1). The second module (F) contains cell cycle regulators (CDK1, CCND1, PLK1), structural proteins (ACTB, FN1), signalling molecules (IL6, CDC42), an adhesion molecule (CDH1) and the ubiquitin conjugating enzyme UBC. These modules highlight distinct functional groupings: one centred on metabolism and stress responses and the other on proliferation and adhesion. The identification of CDK1, ACTB, GAPDH and CDC42 as top hubs suggests that these proteins serve as critical connectors between proliferative, metabolic and structural pathways and may represent potential targets for therapeutic intervention.

## Differentially expressed genes converge on the PI3K-AKT signalling pathway, revealing pathway activation in MCF 7 breast cancer cells:

The KEGG map shows extensive activation of the PI3K-AKT signalling pathway in cancerous MCF 7 cells (Figure 6 - see PDF). Multiple receptor level genes including growth factor receptors and focal adhesion proteins are up and down regulated, suggesting enhanced up and down stream signalling. Central components of the pathway, such as the catalytic and regulatory subunits of phosphatidylinositol 3 kinase (PI3K), phosphoinositide dependent kinase 1 (PDK1) and AKT itself, are highlighted in red, indicating elevated expression. Downstream branches of the pathway also contain numerous up regulated genes: mTOR signalling components (e.g. MTOR, RPTOR), GSK 3β (GSK3B) and FOXO transcription factors (FOXO1/FOXO3) involved in cell cycle control and metabolism are all marked, as are cell cycle regulators (CCND1, CDK1), metabolic enzymes and anti apoptotic proteins (BAD, BCL2). The map further illustrates links to other signalling cascades, including MAPK, HIF 1, p53 and NF κB pathways, underscoring the central role of PI3K-AKT signalling in coordinating proliferation, metabolism and survival. Collectively, the enrichment of up regulated genes throughout the pathway indicates that PI3K-AKT signalling is broadly activated in MCF 7 cells, driving oncogenic processes such as growth, metabolic reprogramming and resistance to apoptosis.

KEGG pathway mapping was performed using Pathview to visualise the distribution of up regulated genes on the PI3K-AKT signalling pathway. The canonical PI3K-AKT pathway is illustrated, with boxes representing individual gene products and arrows denoting signalling relationships. Genes up regulated in MCF 7 relative to MCF 10A are highlighted in red. The pathway integrates inputs from receptor tyrosine kinases and integrins (left), through PI3K activation and PDK1/AKT signalling (centre), to a wide array of downstream effectors governing metabolism, cell cycle progression, growth and survival (right). The present study provides an integrative assessment of how circadian disruption may contribute to breast cancer by comparing transcriptional profiles of MCF-7 and MCF-10A cells. Consistent with evidence that circadian rhythms act as pivotal regulators of breast tumour development [42], we observed widespread differences in gene expression and rhythmicity between the cancerous and non-cancerous lines. A total of 1,788 differentially expressed genes were identified, with roughly balanced numbers of up- and downregulated transcripts. Upregulated genes were strongly enriched for processes such as DNA replication; chromatin organization, mitotic cell cycle and protein localisation, while down regulated genes were enriched for epidermal differentiation, cell-cell adhesion and apoptosis. These complementary patterns suggest that breast cancer cells simultaneously engage oncogenic programmes that promote proliferation and biosynthesis while suppressing differentiation and cell death pathways-a dual strategy that may be fostered by circadian misalignment [42]. Our data implicate the core clock machinery itself in this imbalance. Transcriptional oscillations of BMAL1, CLOCK and PER3 were markedly attenuated in MCF-7 cells and the overall number and amplitude of cycling genes were reduced. Circadian rhythms are known to regulate many physiological processes and their disruption has been linked to tumour initiation and progression in epidemiological and experimental studies [43, 44, 45, 46, 47-48]. The diminished rhythmicity observed here coincided with altered metabolic gene expression, including reduced oxygen consumption and enrichment of glycolysis, gluconeogenesis and carbon metabolism pathways. These findings support the view that circadian dysregulation contributes to the metabolic reprogramming characteristic of cancer [49] echoing reports that interventions targeting clock genes or melatonin signalling can influence breast tumour behaviour [42, 50]. Circadian disruption promotes cancer by impairing metabolism, immunity, DNA repair and cell-cycle control, highlighting therapeutic potential in clock-aligned cancer treatment [51]. In line with previous studies [52, 53], pathway analysis revealed extensive activation of the PI3K-Akt pathway in MCF-7 cells, encompassing upregulation of receptors, PI3K catalytic and regulatory subunits, PDK1, AKT and multiple downstream effectors such as mTOR components, GSK3β, FOXO transcription factors, cell cycle regulators and anti-apoptotic proteins. The PI3K-Akt pathway integrates growth, survival and metabolic signals and its activation has been strongly associated with breast tumour progression and therapeutic resistance [52, 53]. Furthermore, crosstalk with MAPK, HIF-1, p53 and NF-κB pathways was evident, suggesting that circadian disruption may sensitize tumour cells to a wide spectrum of oncogenic cues. Protein-protein interaction network analysis provided further insight into the organisational principles of the tumour transcriptome. The DEGs formed a densely connected network and consensus hub gene analysis across eight centrality metrics converged on ACTB, GAPDH and CDK1 as key hubs. ACTB (β-actin) and GAPDH, despite being traditional housekeeping genes, demonstrated critical involvement in cytoskeletal dynamics and glycolysis, reflecting their broader roles in tumour biology [54, 55]. CDK1, a master regulator of the G2/M transition, also emerged as a prominent hub, consistent with its known importance in breast cancer progression and prognosis [56]. Additional hub genes including FN1, CDC42, IL6, CDH1 and UBC formed two functional modules: one centred on metabolic and stress responses and the other on proliferation and adhesion [57]. These modules highlight how metabolic rewiring, inflammatory signalling and cell cycle control are interlinked in the cancerous state. Downregulated genes were enriched in pathways related to epithelial differentiation, cell adhesion and apoptosis, consistent with an epithelial-mesenchymal transition (EMT)-like phenotype [58, 59]. Suppression of adhesion molecules and apoptotic factors may enable tumour cells to evade cell death and increase invasiveness. This coordinated repression of adhesion and immune-regulatory genes reinforces the malignant phenotype [60]. The integration of circadian disruption, oncogenic signalling and metabolic reprogramming underscores potential therapeutic targets. Interventions aimed at restoring circadian rhythm, such as chronotherapy or pharmacological activation of clock genes, may improve treatment outcomes [61]. Furthermore, targeting PI3K-Akt signalling in combination with circadian-based strategies may synergistically inhibit tumour growth.

## Limitations and future perspectives:

Several limitations warrant consideration. This study is based on a single microarray dataset derived from cell lines [62], which may not capture the full heterogeneity of human breast tumours. Experimental validation including quantitative PCR of clock and hub genes and functional assays assessing the impact of their modulation on proliferation and metabolism-will be essential to confirm these findings. Future studies integrating proteomics, metabolomics and epigenomic data will further elucidate the multidimensional role of circadian regulation in breast cancer. Investigating whether restoration of clock gene expression or targeted inhibition of PI3K-Akt signalling can reverse malignant phenotypes remains a promising avenue.

## Conclusion:

Collectively, our findings provide mechanistic insights into how disrupted circadian regulation drives oncogenic transcriptional programmes and metabolic rewiring in breast cancer. They emphasize the potential of circadian-based interventions and network-targeted therapies to restore homeostasis and improve clinical outcomes in patients. Circadian disruption driven by light-at-night, shift work and sleep irregularity elevates cancer risk through melatonin suppression, CLOCK/BMAL1 dysregulation, impaired immunity, metabolic imbalance and substantial economic burden [63].

## Author contributions:

Yasmin Fatima, Mohammad Kashif and Prashant Ankur Jain conceptualize the manuscript. Yasmin Fatima and Mohammad Kashif performed the experiments, analyzed data, along with preparation of the tables and figures. Yasmin Fatima, Mohammad Kashif and Prashant Ankur Jain wrote original draft and edited the final manuscript.

## Funding: 

No funding

## Data availability statement:

Publicly available datasets were analyzed, taken from GEO datasets (accession number: GSE76370). Since the dataset was already publicly available, this study does not require additional ethics approval.

## ORCID

Mohammad Kashif (0000-0002-1606-2340)

Yasmin Fatima (0000-0001-9367-9443)

## Declarations:

## Consent to Publish declaration:

Pre-PRINT is not published elsewhere

## Consent to Participate declaration:

Not applicable

## Ethics declaration:

Not applicable

## Clinical trial registration:

Not applicable

## Clinical trial number:

Not applicable
